# Gel Containing Catechin and Mesoporous Silica Nanoparticles for Protecting Root Dentin Against Erosion: An In Situ Study

**DOI:** 10.1002/jemt.70042

**Published:** 2025-07-14

**Authors:** Helaine Cajado Alves, Edison Augusto Balreira Gomes, Antonia Flavia Justino Uchoa, Nágila Maria Pontes Silva Ricardo, Vanara Florêncio Passos, Sérgio Lima Santiago

**Affiliations:** ^1^ Graduate Program in Dentistry Faculty of Pharmacy, Dentistry and Nursing, Federal University of Ceará Fortaleza Ceará Brazil; ^2^ Department of Organic and Inorganic Chemistry Federal University of Ceará Fortaleza Ceará Brazil; ^3^ Department of Restorative Dentistry Faculty of Pharmacy, Dentistry and Nursing, Federal University of Ceará Fortaleza Ceará Brazil

**Keywords:** catechin, dentin, drug liberation, matrix metalloproteinase inhibitors, tooth erosion

## Abstract

This study evaluated the in situ anti‐erosive effect of gels containing epigallocatechin‐3‐gallate (EGCG) isolated and adsorbed on mesoporous silica nanoparticles (EGCG/MSN) on eroded dentin. Eleven volunteers participated in this randomized, controlled, cross‐over study, which consisted of 4 phases of 5 days. Acrylic palatal devices were utilized containing two dentin blocks treated with one of the gels: placebo (negative control), SnF_2_ (0.05%—positive control), EGCG (0.1%), and EGCG/MSN (0.093%). The specimens were immersed in citric acid (0.05 M; pH 3.75) for 60 s, 4×/day, followed by treatment with the assigned gel for 60 s. The alterations were evaluated by the percentage of surface hardness loss (%SHL) and through profilometry analysis (wear). Morphological changes were assessed using scanning electron microscopy (SEM). The data were analyzed using ANOVA, followed by Tukey's post‐test. The %SHL did not show a significant difference among the groups. Regarding surface wear, the mean results in micrometers were: placebo, 0.66 (±0.38); EGCG, 0.57 (±0.11); EGCG/MSN, 0.48 (±0.05); and SnF_2_, 0.32 (±0.08). A significant difference was observed between the SnF_2_ group and the placebo and EGCG groups. However, there were no difference between the EGCG/MSN group and the control ones. Within the limitations of the study, EGCG/MSN may act as a protective measure in reducing dentin wear under erosive conditions since it did not differ from the positive control.

## Graphical abstract

Gels containing EGCG adsorbed on mesoporous silica nanoparticles have a protective effect against dentin erosion.

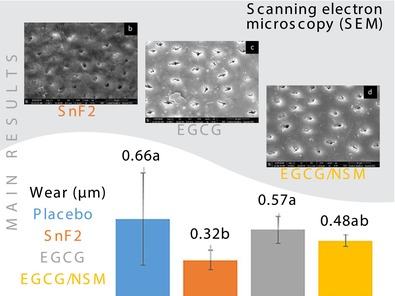

**Summary**

- EGCG/MSN gel preserves the demineralized organic matrix;
- Biomodification agents decreased the effects caused by erosion;
- Micrographs demonstrate the protection of the dentin surface on the EGCG/MSN‐treated.

## 1 Introduction

Erosive tooth wear is a complex condition that affects various age groups within the global population (Leven and Ashley 2023; Nijakowski et al. 2023). It is characterized by the chemical loss of mineralized tooth substance due to exposure to non‐bacterial acids (Jaeggi and Lussi 2014; Schlueter et al. 2020). The prevalence of dental erosion has increased worldwide, largely due to changes in lifestyle and dietary habits, including higher consumption of acidic foods, carbonated beverages, and fresh fruit juices (Chan et al. 2020; Lussi et al. 2011). Prevalence studies have reported erosive conditions in young adults ranging from 7.3% to 33.8% (Vargas‐Ferreira et al. 2011; Bartlett et al. 2013; Skalsky Jarkander et al. 2018; Bartlett and O'Toole 2020).

Controlling erosive acid attacks on dental tissues poses a significant challenge in preventing and treating dental erosion in clinical practice (Bartlett and O'Toole 2020; Donovan et al. 2021). While saliva and remineralizing foods have been shown to decrease enamel erosion (Donovan et al. 2021; Cheaib and Lussi 2021), various measures to reduce tooth wear have been investigated, particularly those that enhance acid resistance or promote the remineralization of tooth surfaces. Traditional preventive and control treatments for dental erosion emphasize patient education, dietary management, and psychological counseling (Donovan et al. 2021). However, some treatments aim to promote mineralization through the application of fluoride. Fluoride treatments facilitate the formation of calcium fluoride on the tooth surface (in the case of amine fluoride and sodium fluoride), which is easily soluble in acid or metal‐rich precipitate formation (in the case of titanium tetrafluoride and fluoride products containing tin) (Donovan et al. 2021).

Among the fluorides studied for their anti‐erosion effects on dentin, those containing tin, a polyvalent metallic cation, have emerged as effective agents (Cheaib and Lussi 2021; Schlueter, Klimek, and Ganss 2011; Passos et al. 2014, 2015; Rabelo et al. 2023). Tin forms a protective barrier on the dentin surface due to its strong affinity for mineralized dental tissue and acts as an inhibitor of matrix metalloproteinases (MMPs) 2 and 9 (Cvikl et al. 2028). However, stannous fluoride may cause undesirable effects, such as staining of the tooth surface and an astringent sensation on the oral mucosa (Donovan et al. 2021; Schlueter, Hara, et al. 2011). Therefore, there is a pressing need to explore more biocompatible substances to minimize the effects of erosion. The use of MMP inhibitors has emerged as a new approach to treating this condition. Green tea and its primary catechin, epigallocatechin‐3‐gallate (EGCG), fall into this category, as they have demonstrated significant reductions in dentin wear after erosive/abrasive challenges along with low toxicity in dental pulp cells (Nakanishi et al. 2010; Vidal et al. 2014; De Moraes et al. 2016; Wang et al. 2018). Additionally, they act as cross‐linking agents for collagen fibrils, stabilizing collagen and improving the mechanical properties of dentin while inhibiting proteolytic degradation of the dentin's organic matrix (Vidal et al. 2014).

In medicine, the use of drug nanocarriers and functional nanoparticles has been extensively explored in cancer treatment. Mesoporous silica nanoparticles (MSNs) are a type of nanocarrier known for their stability, large surface area, and excellent thermal and chemical performance (Chen et al. 2013; Hao et al. 2015; Li et al. 2017; Fang et al. 2023). In dentistry, MSNs have been utilized with nanohydroxyapatite (nHAp), EGCG, calcium oxide, and calcium carbonate, with or without phosphoric acid. In these applications, MSNs serve as carriers for these substances to the dentinal tubules (Chiang et al. 2014; Yu et al. 2016, 2017). Due to challenges in conducting in vivo erosive studies, in situ and in vitro models have been utilized to analyze erosive challenges in dental tissues. In situ studies offer a more realistic evaluation of the effects of erosive agents and protective substances on dental tissues within the oral environment, taking into consideration factors such as salivary flow, acquired pellicle formation, and routine care (West et al. 2011).

The present in situ study aims to evaluate the effects of the application of a gel containing MMP inhibitors, specifically EGCG or EGCG/MSN, on extrinsic erosion caused by citric acid. For this purpose, a placebo and SnF_2_ will be used as control groups, allowing the comparison of the efficacy of the treatments. The null hypothesis tested was that there is no difference among the EGCG isolated or adsorbed on mesoporous silica nanoparticles (EGCG/MSN) and the controls in the protective effect on dentin erosion after extrinsic erosive attack.

## 2 Materials and Methods

### 2.1 Experimental Design

**FIGURE 1:**
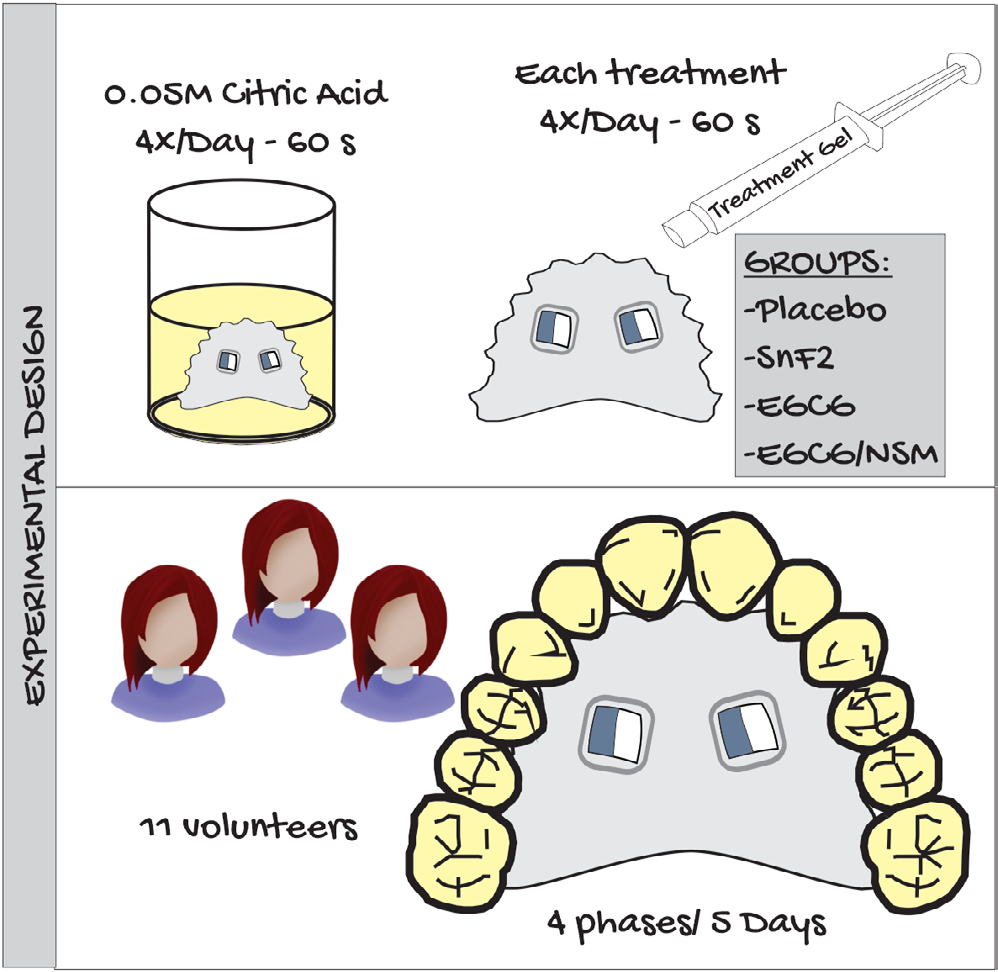
Experimental design.

The present in situ, randomized, controlled, blind, crossover study evaluated the effects of applying a gel containing MMP inhibitors, 0.1% EGCG, or 0.093% EGCG/MSN, on citric acid‐induced extrinsic erosion, using both a placebo and 0.05% SnF_2_ as controls. The experiment consisted of four phases, each lasting 5 days, with a two‐day washout period between phases. Volunteers wore a palatal device containing two dentin blocks (Figure 1). This was immersed four times per day in 50 mL of citric acid solution (0.05 M and pH 3.75) at room temperature for 60 s. After each erosive challenge, the predetermined treatment was applied for 60 s. At the end of each phase, the dentin blocks were removed from the device for quantitative analyses, including hardness and profilometry tests. New dentin blocks were placed in the device to begin a new phase with a different treatment. The response variables were the percentage of surface hardness loss (%SHL), the wear profile of the dentin structure, and the surface morphology analyzed by SEM.

### 2.2 Reagents

The following reagents were used: EGCG, hexadecyltrimethylammonium bromide (CTAB), ammonium hydroxide (NH_4_OH), tetraethyl orthosilicate (TEOS) and ammonium nitrate (NH_4_NO_3_), SnF_2_, PLURONIC F127 from Sigma‐Aldrich (St. Louis, MO, USA). All reagents were of analytical grade and used without prior purification.

### 2.3 Synthesis of MSNs

The MSNs were synthesized following a previously reported method with slight modifications (Zou et al. 2017). First, 5.73 g of CTAB were dissolved in 280 mL of deionized water under mechanical stirring at 40°C for 15 min and then kept stirring overnight at room temperature. After this period, 0.5 mL of NH4OH and 80 mL of ethanol were added to the solution, which was stirred for an additional hour. The temperature was then raised to 70°C, and 14.6 mL of TEOS was added dropwise. The reaction mixture was stirred for a further 2 h. Subsequently, the resulting solid was collected by centrifugation, washed with ethanol, and dried at 100°C for 5 h. Surfactant extraction was performed using an NH_4_NO_3_/ethanol mixture (133 mg/50 mL) at 60°C, with continuous magnetic stirring for 40 min. Finally, the solid was collected by centrifugation, washed three times with deionized water, and dried at 100°C overnight, thus obtaining the MSNs.

### 2.4 Synthesis of Epigallocatechin‐3‐Gallate Adsorbed on Mesoporous Silica Nanoparticles (EGCG/MSN)

The synthesis of EGCG‐loaded mesoporous silica nanoparticles (EGCG/MSN) was carried out following the method described by Yu et al. (Yu et al. 2017), with minor modifications. Briefly, 100 mg of dry MSN were dispersed in an aqueous EGCG solution (1.8 mg/mL) under vigorous horizontal stirring for 72 h in the dark. This process allowed the EGCG molecules to infiltrate the pores of the MSNs, achieving maximum loading. The mixture was then centrifuged, washed three times with deionized water and ethanol, and dried under vacuum for 48 h to obtain EGCG/MSN, being stored in the dark at 4°C until use.

To quantify the drug loading, a calibration curve was prepared using a series of reference solutions with concentrations ranging from 0.1 to 30 ppm, resulting in a linear correlation between the maximum absorbance peak and the EGCG concentration. A UV‐Vis spectrophotometer (DU 730; Beckman Coulter, Fullerton, CA, USA) was used to assess and confirm the peak absorbance of EGCG at 274 nm. The encapsulation efficiency was calculated at 93%.

### 2.5 Gel Synthesis

The copolymer PLURONIC F127 (Sigma Chemical Co., St. Louis, MO, USA) was used to prepare both placebo and active gels, containing the following mass percentages: no active component (placebo), 0.05% of SnF_2_, 0.1% of EGCG, or 37% of EGCG/MSN. The neutral gel base was prepared at a concentration of 20% PLURONIC F127/water. Specifically, 6 g of PLURONIC F127 were dissolved in 30 mL of deionized water at low temperature (approximately 4°C) under magnetic stirring until complete homogenization was achieved. Once fully dissolved, the temperature was raised to 30°C to induce gelation.

### 2.6 Ethical Aspects and Volunteers Selection

Before the start of this study, the research protocol was approved by the local Ethics Committee (approval no. 2.704.870) and conducted in accordance with the principles of the Declaration of Helsinki. All volunteers previously signed a written Informed Consent form and received both verbal and written instructions regarding the study procedures.

A total of 11 volunteers (1 male and 10 females), aged between 22 and 41 years, were recruited for the study. All the selected volunteers met the following inclusion criteria: good oral hygiene, absence of caries activity and periodontal disease, no signs of erosive lesions, and stimulated salivary flow greater than 1 mL/min. Exclusion criteria included the presence of gastroesophageal disorders, pregnant women, breastfeeding, use of a myorelaxant night guard, fixed or removable orthodontic appliances in the upper arch, or any medications capable of altering salivary flow.

The sample size was calculated based on percentage changes in surface hardness loss data obtained from a previous study (De Moraes et al. 2016). A minimum of 10 volunteers was required to achieve an α‐error of 5%, 80% statistical power, and a detectable difference of 0.82 between groups (calculated using OpenEpi; www.openepi.com/menu/oe_menu.htm). To compensate for potential dropouts inherent to in situ studies, 15 volunteers were recruited; however, only 11 completed the experiment.

### 2.7 Preparation of Dentin Specimens

In this study, freshly extracted, non‐carious human third molars were collected after informed consent was obtained from the donors, following a protocol approved by the Ethics Committee of the Federal University of Ceará, Brazil. The selected teeth were cleaned and stored in a 0.1% thymol solution at 4°C until use. Approximately 200 human root dentin blocks were sectioned (4 × 4 × 2 mm), flattened, and polished. A double‐sided diamond cutting wheel (Struers, Minitom, Copenhagen, Denmark) under abundant refrigeration was used for sectioning. The dentin blocks were then placed in an acrylic device and sequentially flattened using P1200, P2500, and P4000 aluminum oxide abrasive sandpapers (Buehler, Lake Bluff, IL, USA), coupled to an automatic polisher (Buehler, Automet 250, Lake Bluff, IL, USA) under continuous water irrigation, followed by polishing with a felt disc and 1 μm diamond paste (Erios, São Paulo, Brazil).

During polishing, the specimens were sonicated in distilled water for 2 min both between each abrasive step and again after final polishing to remove any residual debris. The initial surface hardness was determined to standardize the specimens. Five indentations, spaced 100 μm apart, were made in the center of each specimen using a microhardness tester with a Knoop indenter and automatic measuring system (Future Tech Corp, FM‐ARS 9000 and FM 100, Tokyo, Japan). The indentations were made using a load of 10 g applied for 5 s (De Moraes et al. 2016; Moraes et al. 2021). A total of 88 dentin specimens, with a mean surface hardness of 54.53 ± 5.4 kg/mm^2^, were selected, packaged, identified, and sterilized using ethylene oxide. Each specimen was partially covered with acid‐resistant adhesive tape, leaving an exposed area of 4 × 2 mm that was subjected to erosion and treatment during the procedures. The covered area served as a reference for the profilometric analysis.

### 2.8 Palatal Device Preparation

For each volunteer, an acrylic resin palatal device was custom‐fabricated. Two cavities measuring 5 × 5 × 3 mm were created on the right and left sides of the device. Specimens were randomly allocated using a computer‐generated list (Microsoft Excel 2007) and fixed in place with sticky wax, ensuring they were positioned 1 mm below the surface level of the device to prevent mechanical abrasion resulting from tongue contact.

### 2.9 Intraoral Phase

Two days prior to the start of the experiment, volunteers began using a standard fluoride toothpaste (Colgate‐1450 ppm fluoride), which was provided and used throughout the study. Before each experimental phase, the palatal device was worn for 12 h to allow equilibrium with the saliva and the formation and maturation of an acquired pellicle. A crossover design was employed, with all volunteers participating in all stages and testing all treatment conditions. Specimen positions were randomized using a computer‐generated allocation list.

The erosive challenge was performed extraorally, four times daily at 7:00 am, 12:00 pm, 5:00 pm, and 9:00 pm, over a 5‐day period (Passos et al. 2017). Participants removed the device and immersed it in 50 mL of citric acid solution (0.05 M, pH 3.75) for 60 s at room temperature to simulate erosive conditions (De Moraes et al. 2016). After each immersion, they washed the device under running water for 10 s, and excess moisture was gently removed with absorbent paper. Subsequently, a thin layer of the assigned gel was applied to the dentin surface and left in contact for 60 s before excess gel was removed with absorbent paper (Figure 1). After that, the device was reinserted into the mouth. The gels were coded and dispensed in syringes to maintain the blindness of the study regarding the volunteers.

Volunteers were instructed to avoid eating, drinking (except water), and brushing their teeth while wearing the device, which was used continuously, including at night, and was only removed for meals and oral hygiene. During these intervals, the device was kept mostly in plastic containers. Volunteers were permitted to clean the device with a toothbrush and toothpaste, taking care to avoid brushing the specimens.

At the end of each experimental phase, on the morning of the sixth day, the specimens were removed from the device and stored in a humid environment under refrigeration until the time of analysis. They were evaluated with respect to the percentage of surface hardness loss (%SHL) and the measurement of dentin surface wear. New specimens were inserted into the device to begin the next experimental phase.

### 2.10 Final Surface Hardness Assessment

The final surface hardness of each specimen was measured using the same protocol as the initial surface hardness. Five indentations, spaced 100 μm apart, were made at the center of the eroded area on each specimen. The mean value of these indentations (final hardness) was used to evaluate the percentage loss of surface hardness (%SHL) according to the following formula:

$$\%\mathrm{SHL}=\left[\left(\text{initial hardness}-\text{final hardness}\right)\times 100/\text{initial hardness}\right]$$

### 2.11 Assessment of Dentin Wear

The level of dentin wear was determined in relation to the reference area with a profilometer (Hommel Tester, T1000, Hommelwerke GmbH, Germany). After carefully removing the adhesive from each dentin block to expose the reference area, three tracings, each 1.5 mm in length, were performed on each specimen. The profilometer pen tip moved 1.8 μm from the reference surface to the experimental surface. The range of measurement of the tip is from ±80 μm with a resolution of 0.01 μm. The average of the three tracings measurements was recorded for each specimen.

### 2.12 Scanning Electron Microscopy

**FIGURE 2:**
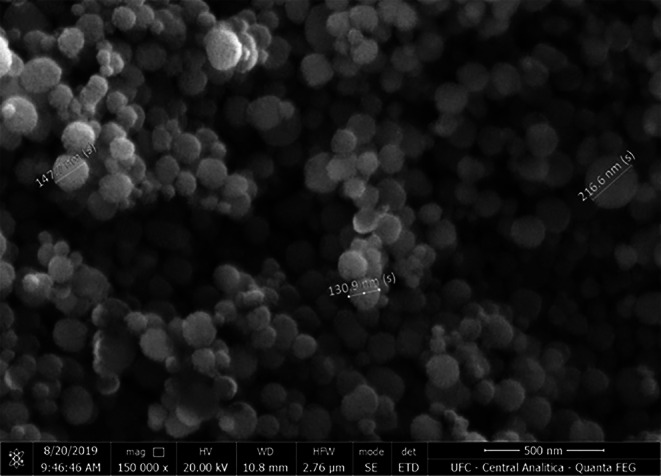
Scanning electron micrographs (150.000×) of mesoporous silica nanoparticles with EGCG (average size 164 nm).

Two dentin samples from each group were analyzed using scanning electron microscopy (SEM) for qualitative analysis. The samples were immersed for fixation in a 2.5% glutaraldehyde solution in a 0.1 mol/L sodium cacodylate for 24 h, followed by rinsing with 0.1 mol/L cacodylate buffer. Next, the samples were dehydrated with ethanol solutions of increasing concentrations and dried at room temperature for 24 h in a desiccator (Toledano et al. 2012). The specimens were mounted on metal stubs and coated with gold through a metallizer (Hammer VI—sputtering system, Anatech Ltda, Alexandria, USA). After coating, the samples were examined using a Quanta FEG 450 SEM (FEI Company, Oregon, USA) with an accelerating voltage of 20 kV and a magnification of 8000×. Additionally, SEM was used to observe the morphology and ultrastructure of MSN. The (EGCG/MSN) gel was also qualitatively analyzed by SEM to verify its adsorption (Figure 2).

### 2.13 Statistical Analysis

The mean and standard deviation for wear and hardness loss were calculated. The normality of data distribution was assessed using the Kolmogorov–Smirnov test. Since all groups showed normal distribution, one‐way ANOVA and Tukey post hoc tests were applied for the data of wear and hardness loss. Statistical analysis was conducted using the Statistical Package for Social Sciences (SPSS) version 20.0 for Windows (SPSS Inc., Chicago IL, USA). The significance level was set at 5%.

## 3 Results

**FIGURE 3:**
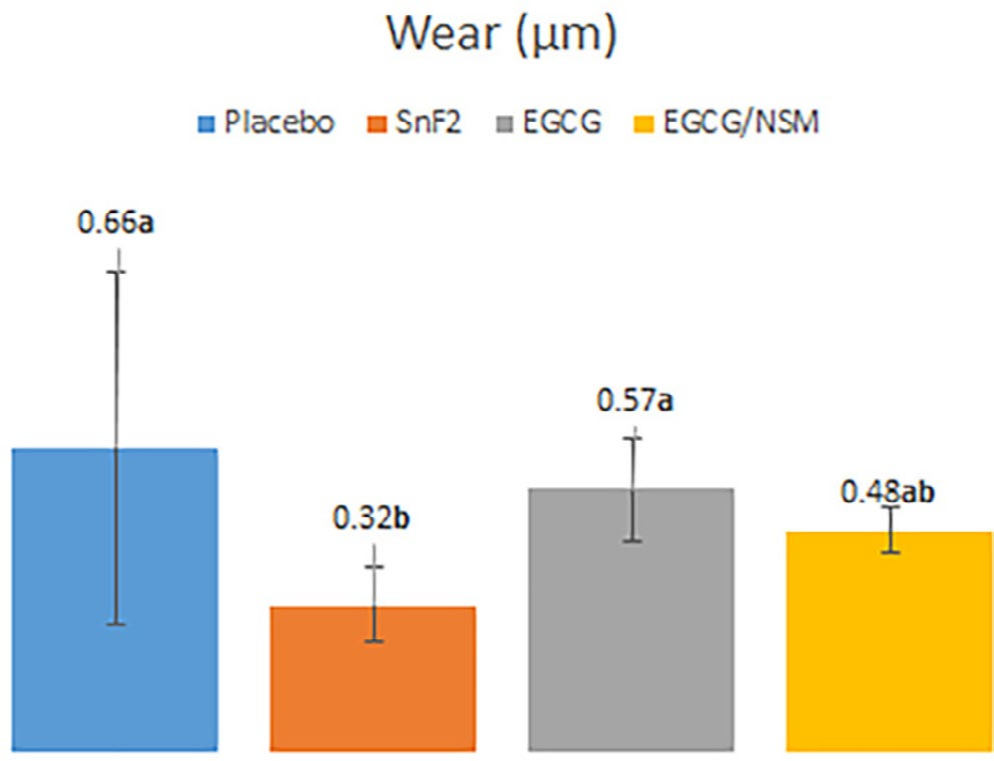
Mean values of wear (μm). Vertical bars and lines denote wear differences in the groups studied and the standard deviations, respectively. Different letters denote statistically significant differences identified via Tukey test.

The mean and standard deviation values of dentin surface hardness before (initial) and after (final) the erosive challenge/treatment, as well as the percentage of surface hardness loss (%SHL) are presented in Table 1. The %SHL analysis showed no statistically significant difference between the groups (*p* = 0.067). In contrast, dentin wear values (μm) revealed a statistically significant difference between treatments (*p* = 0.005). Therefore, the Tukey post‐test was applied to determine the difference between the groups, which were observed only for SnF_2_ when compared to the placebo and EGCG groups (*p* = 0.003 and *p* = 0.046, respectively). Regarding the EGCG/MSN group, no statistically significant difference was observed compared to SnF_2_ (positive control) (*p* = 0.306), as shown in Figure 3.

**TABLE 1:** Mean (*n* = 11; SD) of dentin surface hardness (SH) before (baseline), after the erosive challenge/gel treatments, and percentage of loss (%SHL).

Groups	Dentin surface hardness		
	Baseline	Final	%SHL
Placebo	56.17 (3.54)	39.22 (7.55)	30.17 (16.24)
SnF_2_	56.14 (2.17)	43.15 (3.56)	23.13 (6.52)
EGCG	56.09 (2.91)	41.25 (3.38)	26.45 (8.53)
EGCG/MSN	55.28 (3.39)	45.87 (6.28)	17.02 (8.86)
*p*	0.887	0.052	0.067

**FIGURE 4:**
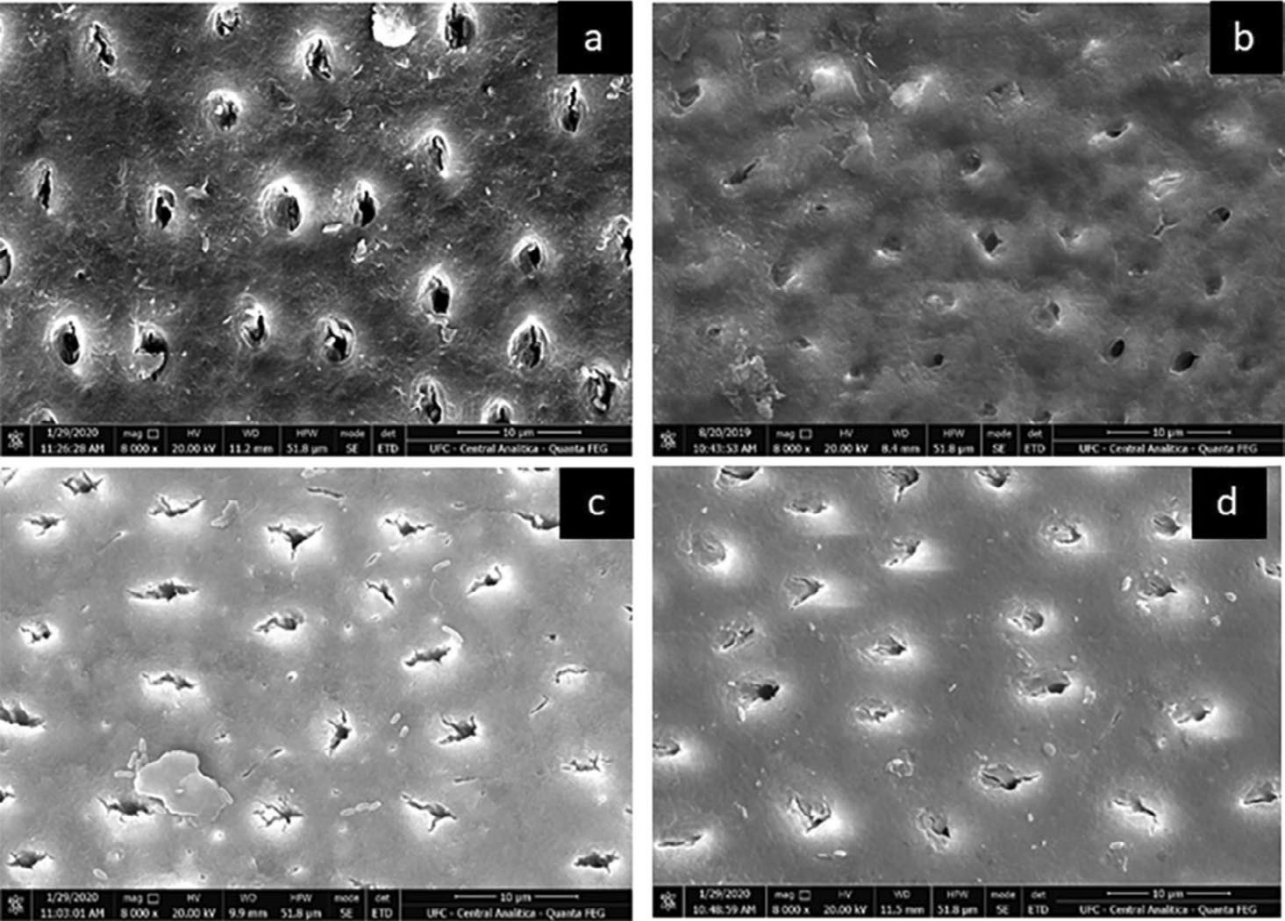
SEM (×8000): (a) placebo, (b) EGCG, (c) SnF_2_, (d) EGCG/MSN.

Scanning electron microscopy (SEM) images showed the patent dentinal tubules in the negative control group (placebo). In contrast, specimens treated with EGCG and EGCG/MSN exhibited the patent tubules obliterated, similar to the SnF_2_ group (Figure 4).

### 3.1 Discussion

The present study aimed to evaluate the effect of applying a gel containing MMP inhibitors (EGCG, EGCG/MSN) on the protection of human root dentin against erosion induced by citric acid. The evaluation was conducted using an in situ model to simulate clinical conditions, such as using human dentin substrate, the natural formation of acquired pellicle, and physiological salivary flow (West et al. 2011).

The quantitative methods selected to analyze dentin surface alterations after erosive challenges were surface hardness measurement and profilometric analysis. While hardness measurement is commonly used to assess changes in enamel surface in the early stages of erosion (Shellis et al. 2011), it was chosen in this study because the erosion protocol employed citric acid (0.05 M and pH 3.75), which has a lower erosive potential compared to other extrinsic agents such as a Coca‐Cola (pH 2.6), used in other studies (Wang et al. 2018; Kato, Leite, Hannas, and Buzalaf 2010; Magalhães et al. 2009). However, a limitation of this method is that in severely eroded dental substrates, where indentation limits are not clearly defined, measurements can become imprecise or even impossible (Schlueter, Hara, et al. 2011). This limitation may partly explain the absence of statistical differences between groups. For this reason, profilometric analysis was also performed, as it is a widely validated method for assessing dentin wear after erosive challenges (Schlueter, Hara, et al. 2011; Shellis et al. 2011; Kato, Leite, Hannas, and Buzalaf 2010; Magalhães et al. 2009; Passos et al. 2018; Bueno et al. 2022). However, the outcomes failed to reject the null hypothesis since there is no difference between tested groups and controls.

Furthermore, all tested products used a gel as a delivery vehicle for the substances to increase the contact time with dentin, enhancing the substantivity of the active ingredient and making clinical use more practical. Because the tested gels shared identical formulations, any observed preventive impact on erosion could be attributed to the active compounds. Previous studies using gel as a vehicle in delivering MMP inhibitors (EGCG and FeSO_4_) have demonstrated their effectiveness in reducing tooth wear (Kato, Leite, Hannas, and Buzalaf 2010; Kato, Leite, Hannas, Oliveira, et al. 2010; Kato et al. 2021). An additional strategy to increase contact time with the dentin substrate was the encapsulation of EGCG in MSNs (nanoscale microcapsules), allowing for continuous drug release. The use of MSNs for drug encapsulation has shown promising results in Dentistry, including dentinal tubule occlusion and the introduction of chlorhexidine in glass ionomer cement (Fang et al. 2023; Yu et al. 2017; Yan et al. 2017). To the best of the authors' knowledge, no studies evaluated the use of EGCG/MSN for protection against dentin erosion.

MMP inhibitors, such as the catechins found in green tea, particularly EGCG, have shown efficacy in preserving the demineralized organic matrix (DOM) (Kato et al. 2012). EGCG has been identified as an inhibitor of MMPs 2 and 9, with minimum inhibitory concentration values of 6 and 0.8 μM, respectively (Demeule et al. 2000). Therefore, the concentration used in this current study (0.1% equivalent to 4000 μM) was considered sufficient to inhibit these collagenases, as shown in previous research (Moraes et al. 2021). However, in the present study, dentin wear was similar for the EGCG gel and the negative control. Likewise, another in situ study showed that the EGCG gel was unable to prevent tooth tissue loss after erosive challenges (Coca‐Cola, 1 min; 4 times/day/5 days) (Moraes et al. 2021). In contrast, previous studies (Kato, Leite, Hannas, and Buzalaf 2010; Kato, Leite, Hannas, Oliveira, et al. 2010) demonstrated that the EGCG gel significantly reduced dentin wear compared to the negative control. This discrepancy might be explained by the fact that the specimens in the current study underwent 12 h of use to allow the formation of acquired pellicle, which serves as a natural protective barrier against erosion, although its effect is more pronounced on enamel than on dentin (Wiegand et al. 2008). Although a validated methodology was employed in this study, subjecting the samples to a more rigorous erosion challenge could have improved the ability to detect potential differences among the groups. Several in vitro studies (De Moraes et al. 2016; Passos et al. 2018; Leal et al. 2021) reported that green tea and/or EGCG reduced dentin wear, differing from the negative control. However, it is important to note that in all these studies, treatments were applied for 5 min, whereas in the present study, application lasted only 1 min, which may account for the absence of significant differences.

One factor that seems to influence the effectiveness of MMP inhibitors is the timing of application. In situ studies by Kato et al. (Kato, Leite, Hannas, and Buzalaf 2010) and Kato et al. (Kato, Leite, Hannas, Oliveira, et al. 2010) found that applying protease inhibitors prior to the erosive challenge almost completely inhibited dentin wear (around 0.05 μm). In the present study, the application was performed for the same duration of time, and after the erosion process, resulting in dentin wear values of 0.57 μm for EGCG and 0.48 μm for EGCG/MSN. Kato et al. (Kato et al. 2009) reported similar dentin wear values (0.59 μm) when EGCG at a concentration of 400 μm was applied for 1 min after erosion (Kato et al. 2009). These findings suggest that applying MMP inhibitors before erosion may yield better results, likely due to their interaction with inactive collagenases in the dentin, which prevents their activation during subsequent pH reductions. MMPs require a drop in pH for activation, followed by medium neutralization, before they can degrade the DOM (Buzalaf et al. 2012; Chaussain‐Miller et al. 2006).

It is believed that the comparable performance of EGCG/MSN and SnF_2_ in reducing dentin wear is related to the characteristics of MSNs, which are resistant to acid challenge (Li et al. 2011) and capable of promoting continuous release of the drug (Yu et al. 2017; Yan et al. 2017). This allows for prolonged contact between catechin and dentin while protecting EGCG from the harsh conditions of the oral environment. Moreover, the gels were applied four times a day, which exceeds typical clinical use. This intensified application was intentionally adopted to ensure that any potential differences between the tested groups could be accurately detected under the mild erosive conditions of the study. This approach, however, represents a limitation, as it may overestimate the real‐world effectiveness of the gels. Additionally, SEM images showed that the surface morphology of the EGCG/MSN‐treated group was similar to that of the SnF_2_ group (Figure 4).

The anti‐erosion effect of tin has been demonstrated in numerous studies (Schlueter et al. 2020; Rabelo et al. 2023; West et al. 2021), supporting the results obtained for SnF_2_ (positive control) in the present study. SnF_2_ has also been identified as an inhibitor of MMPs 2 and 9 (Cvikl et al. 2028), and it protects the DOM by incorporating into mineralized tissue (Ganss et al. 2010). Upon reacting with hydroxyapatite, SnF_2_ forms complex precipitates such as Sn_2_OHPO_4_, Sn_3_F_3_PO_4_, Ca (SnF_3_)_2_, and CaF_2_ (Babcock et al. 1978). The combined action of these mechanisms explains the superior performance of SnF_2_ in reducing dentin wear. The results show that the gel containing SnF_2_ had excellent performance, reducing dentin loss by approximately 51% compared to the placebo gel. However, despite its effectiveness, products containing tin may cause tooth surface discoloration and an astringent sensation (Ellingsen et al. 1982), restricting their practical application in a clinical setting. This highlights the need for natural products, low cost, with easy access and fewer side effects.

Despite employing a well‐established pH‐cycling protocol and striving to closely simulate clinical conditions, this study has limitations. These include the inability to assess the effects of protease inhibitors on salivary MMPs and the lack of a combined evaluation of erosion and abrasion. Moreover, the protocol used in this study did not directly evaluate the inhibitory action of EGCG on MMP. The reduction in dentin wear observed for EGCG/MSN could be attributed both to the action of EGCG and the specific properties of the MSNs. Nevertheless, further studies are needed to confirm the MMP‐inhibitory action of EGCG when adsorbed on MSNs in erosively demineralized dentin.

## 4 Conclusion

It is concluded that, within the limitations of the present study, the use of EGCG/MSN showed a protective effect on dentin similar to SnF_2_, highlighting the potential of this delivery system in reducing dentin erosion. Further studies are needed to confirm its mechanism of action and clinical applicability.

## Author Contributions

**Helaine Cajado Alves:** conceptualization, investigation, writing – original draft, methodology, writing – review and editing, formal analysis, project administration. **Edison Augusto Balreira Gomes:** writing – original draft, methodology, writing – review and editing. **Antonia Flavia Justino Uchoa:** conceptualization, investigation, writing – original draft, writing – review and editing, methodology, formal analysis. **Nágila Maria Pontes Silva Ricardo:** conceptualization, investigation, methodology, validation, writing – review and editing, formal analysis. **Vanara Florêncio Passos:** conceptualization, investigation, writing – original draft, methodology, formal analysis, project administration, supervision, writing – review and editing. **Sérgio Lima Santiago:** conceptualization, investigation, writing – original draft, methodology, writing – review and editing, project administration, supervision.

## Ethics Statement

The present study followed the Declaration of Helsinki for the ethical principles of medical research involving human subjects and was approved by our institute's research ethics committee (approval number 2.704.870).

## Consent

Informed written consent was obtained from all subjects and/or their legal guardians.

## Conflicts of Interest

The authors received no specific funding for this work.

## Data Availability

The data that support the findings of this study are openly available in Research square at https://www.researchsquare.com/article/rs-3996730/v1, reference number https://doi.org/10.21203/rs.3.rs-3996730/v1.

## References

[jemt70042-bib-0001] Babcock, F. D. , J. C. King , and T. H. Jordan . 1978. “The Reaction of Stannous Fluoride and Hydroxyapatite.” Journal of Dental Research 57, no. Suppl 9–10: 933–938. 10.1177/00220345780570092301. 281374

[jemt70042-bib-0003] Bartlett, D. W. , A. Lussi , N. X. West , P. Bouchard , M. Sanz , and D. Bourgeois . 2013. “Prevalence of Tooth Wear on Buccal and Lingual Surfaces and Possible Risk Factors in Young European Adults.” Journal of Dentistry 41, no. Suppl 11: 1007–1013. 10.1016/j.jdent.2013.08.018. 24004965

[jemt70042-bib-0002] Bartlett, D. , and S. O'Toole . 2020. “Tooth Wear and Aging.” Australian Dental Journal 64, no. Suppl 1: 59–62. 10.1111/adj.12681. 31144323

[jemt70042-bib-0004] Bueno, T. L. , T. A. da Silva , F. A. Rizzante , A. C. Magalhães , D. Rios , and H. M. Honório . 2022. “Evaluation of Proanthocyanidin‐Based Dentifrices on Dentin‐Wear After Erosion and Dental Abrasion – In Situ Study.” Journal of Clinical and Experimental Dentistry 14, no. Suppl 4: e366–e370. 10.4317/jced.59071. 35419173 PMC9000390

[jemt70042-bib-0005] Buzalaf, M. A. , M. T. Kato , and A. R. Hannas . 2012. “The Role of Matrix Metalloproteinases in Dental Erosion.” Advances in Dental Research 24, no. Suppl 2: 72–76. 10.1177/0022034512455029. 22899684

[jemt70042-bib-0006] Chan, A. S. , T. T. K. Tran , Y. H. Hsu , S. Y. S. Liu , and J. Kroon . 2020. “A Systematic Review of Dietary Acids and Habits on Dental Erosion in Adolescents.” International Journal of Paediatric Dentistry 30, no. Suppl 6: 713–733. 10.1111/ipd.12643. 32246790

[jemt70042-bib-0007] Chaussain‐Miller, C. , F. Fioretti , M. Goldberg , and S. Menashi . 2006. “The Role of Matrix Metalloproteinases (MMPs) in Human Caries.” Journal of Dental Research 85, no. Suppl 1: 22–32. 10.1177/154405910608500104. 16373676

[jemt70042-bib-0008] Cheaib, Z. , and A. Lussi . 2021. “Impact of Acquired Enamel Pellicle Modification on Initial Dental Erosion.” Caries Research 45, no. Suppl 2: 107–112. 10.1159/000324803. 21412002

[jemt70042-bib-0009] Chen, Y. , H. Chen , and J. Shi . 2013. “In Vivo Bio‐Safety Evaluations and Diagnostic/Therapeutic Applications of Chemically Designed Mesoporous Silica Nanoparticles.” Advanced Materials 25, no. Suppl 23: 3144–3176. 10.1002/adma.201205292. 23681931

[jemt70042-bib-0010] Chiang, Y. C. , H. P. Lin , H. H. Chang , et al. 2014. “A Mesoporous Silica Biomaterial for Dental Biomimetic Crystallization.” ACS Nano 8, no. Suppl 12: 12502–12513. 10.1021/nn5053487. 25482513

[jemt70042-bib-0011] Cvikl, B. , A. Lussi , T. S. Carvalho , A. Moritz , and R. Gruber . 2028. “Stannous Chloride and Stannous Fluoride Are Inhibitors of Matrix Metalloproteinases.” Journal of Dentistry 78: 51–58. 10.1016/j.jdent.2018.08.002. 30081053

[jemt70042-bib-0012] De Moraes, M. D. , J. R. Carneiro , V. F. Passos , and S. L. Santiago . 2016. “Effect of Green Tea as a Protective Measure Against Dental Erosion in Coronary Dentine.” Brazilian Oral Research 30: S1806‐83242016000100213. 10.1590/1807-3107BOR-2016.vol30.0013. 26676195

[jemt70042-bib-0013] Demeule, M. , M. Brossard , M. Pagé , D. Gingras , and R. Béliveau . 2000. “Matrix Metalloproteinase Inhibition by Green Tea Catechins.” Biochimica et Biophysica Acta 1478, no. Suppl 1: 51–60. 10.1016/s0167-4838(00)00009-1. 10719174

[jemt70042-bib-0014] Donovan, T. , C. Nguyen‐Ngoc , I. Abd Alraheam , and K. Irusa . 2021. “Contemporary Diagnosis and Management of Dental Erosion.” Journal of Esthetic and Restorative Dentistry 33, no. Suppl 1: 78–87. 10.1111/jerd.12706. 33410255

[jemt70042-bib-0015] Ellingsen, J. E. , H. M. Eriksen , and G. Rölla . 1982. “Extrinsic Dental Stain Caused by Stannous Fluoride.” Scandinavian Journal of Dental Research 90, no. Suppl 1: 9–13. 10.1111/j.1600-0722.1982.tb01518.x. 6952551

[jemt70042-bib-0016] Fang, L. , H. Zhou , L. Cheng , Y. Wang , F. Liu , and S. Wang . 2023. “The Application of Mesoporous Silica Nanoparticles as a Drug Delivery Vehicle in Oral Disease Treatment.” Frontiers in Cellular and Infection Microbiology 13: 1124411. 10.3389/fcimb.2023.1124411. 36864881 PMC9971568

[jemt70042-bib-0017] Ganss, C. , M. Hardt , A. Lussi , A. K. Cocks , J. Klimek , and N. Schlueter . 2010. “Mechanism of Action of Tin‐Containing Fluoride Solutions as Anti‐Erosive Agents in Dentine ‐ An in Vitro Tin‐Uptake, Tissue Loss, and Scanning Electron Microscopy Study.” European Journal of Oral Sciences 118, no. Suppl 4: 376–384. 10.1111/j.1600-0722.2010.00742.x. 20662911

[jemt70042-bib-0018] Hao, X. , X. Hu , C. Zhang , et al. 2015. “Hybrid Mesoporous Silica‐Based Drug Carrier Nanostructures With Improved Degradability by Hydroxyapatite.” ACS Nano 9, no. Suppl 10: 9614–9625. 10.1021/nn507485j. 26316321

[jemt70042-bib-0019] Jaeggi, T. , and A. Lussi . 2014. “Prevalence, Incidence and Distribution of Erosion.” Monographs in Oral Science 25: 55–73. 10.1159/000360973. 24993258

[jemt70042-bib-0024] Kato, M. T. , A. C. Magalhães , D. Rios , A. R. Hannas , T. Attin , and M. A. Buzalaf . 2009. “Protective Effect of Green Tea on Dentin Erosion and Abrasion.” Journal of Applied Oral Science 17, no. Suppl 6: 560–564. 10.1590/s1678-77572009000600004. 20027426 PMC4327513

[jemt70042-bib-0021] Kato, M. T. , A. L. Leite , A. R. Hannas , and M. A. Buzalaf . 2010. “Gels Containing MMP Inhibitors Prevent Dental Erosion in Situ.” Journal of Dental Research 89, no. Suppl 5: 468–472. 10.1177/0022034510363248. 20200409

[jemt70042-bib-0023] Kato, M. T. , A. L. Leite , A. R. Hannas , et al. 2010. “Effect of Iron on Matrix Metalloproteinase Inhibition and on the Prevention of Dentine Erosion.” Caries Research 44, no. Suppl 3: 309–316. 10.1159/000315932. 20551644

[jemt70042-bib-0022] Kato, M. T. , A. L. Leite , A. R. Hannas , et al. 2012. “Impact of Protease Inhibitors on Dentin Matrix Degradation by Collagenase.” Journal of Dental Research 91, no. Suppl 12: 1119–1123. 10.1177/0022034512455801. 23023765

[jemt70042-bib-0020] Kato, M. T. , A. R. Hannas , C. A. B. Cardoso , et al. 2021. “Dentifrices or Gels Containing MMP Inhibitors Prevent Dentine Loss: In Situ Studies.” Clinical Oral Investigations 25, no. Suppl 4: 2183–2190. 10.1007/s00784-020-03530-y. 32975705

[jemt70042-bib-0025] Leal, I. C. , C. S. Rabelo , Í. E. L. Viana , T. Scaramucci , S. L. Santiago , and V. F. Passos . 2021. “Hesperidin Reduces Dentin Wear After Erosion and Erosion/Abrasion Cycling in Vitro.” Archives of Oral Biology 129: 105208. 10.1016/j.archoralbio.2021.105208. 34298255

[jemt70042-bib-0026] Leven, A. J. , and M. Ashley . 2023. “Epidemiology, Aetiology and Prevention of Tooth Wear.” British Dental Journal 234, no. Suppl 6: 439–444. 10.1038/s41415-023-5624-0. 36964373

[jemt70042-bib-0027] Li, S. , M. Liu , and L. Sun . 2011. “Preparation of Acid‐Resisting Ultramarine Blue by Novel Two‐Step Silica Coating Process.” Industrial & Engineering Chemistry Research 50: 7326–7331. 10.1021/ie200343k.

[jemt70042-bib-0028] Li, Y. , N. Li , W. Pan , Z. Yu , L. Yang , and B. Tang . 2017. “Hollow Mesoporous Silica Nanoparticles With Tunable Structures for Controlled Drug Delivery.” ACS Applied Materials & Interfaces 9, no. Suppl 3: 2123–2129. 10.1021/acsami.6b13876. 28004570

[jemt70042-bib-0029] Lussi, A. , N. Schlueter , E. Rakhmatullina , and C. Ganss . 2011. “Dental Erosion – An Overview With Emphasis on Chemical and Histopathological Aspects.” Caries Research 45, no. Suppl 1: 2–12. 10.1159/000325915. 21625128

[jemt70042-bib-0030] Magalhães, A. C. , A. Wiegand , D. Rios , A. Hannas , T. Attin , and M. A. Buzalaf . 2009. “Chlorhexidine and Green Tea Extract Reduce Dentin Erosion and Abrasion in Situ.” Journal of Dentistry 37, no. Suppl 12: 994–998. 10.1016/j.jdent.2009.08.007. 19733206

[jemt70042-bib-0031] Moraes, M. D. R. D. E. , V. F. Passos , G. C. Padovani , L. C. B. da Rocha Bezerra , I. M. Vasconcelos , and S. L. Santiago . 2021. “Protective Effect of Green Tea Catechins on Eroded Human Dentin: An In Vitro/in Situ Study.” Brazilian Oral Research e108. 10.1590/1807-3107bor-2021.vol35.0108. 34816896

[jemt70042-bib-0032] Nakanishi, T. , K. Mukai , H. Yumoto , K. Hirao , Y. Hosokawa , and T. Matsuo . 2010. “Anti‐Inflammatory Effect of Catechin on Cultured Human Dental Pulp Cells Affected by Bacteria‐Derived Factors.” European Journal of Oral Sciences 118, no. Suppl 2: 145–150. 10.1111/j.1600-0722.2010.00714.x. 20487003

[jemt70042-bib-0033] Nijakowski, K. , J. Jankowski , D. Gruszczyński , and A. Surdacka . 2023. “Eating Disorders and Dental Erosion: A Systematic Review.” Journal of Clinical Medicine 12, no. Suppl 19: 6161. 10.3390/jcm12196161. 37834805 PMC10573129

[jemt70042-bib-0034] Passos, V. F. , A. A. de Vasconcellos , J. H. Pequeno , L. K. Rodrigues , and S. L. Santiago . 2015. “Effect of Commercial Fluoride Dentifrices Against Hydrochloric Acid in an Erosion‐Abrasion Model.” Clinical Oral Investigations 19, no. Suppl 1: 71–76. 10.1007/s00784-014-1213-6. 24578231

[jemt70042-bib-0037] Passos, V. F. , L. K. Rodrigues Gerage , and S. L. Santiago . 2017. “Magnesium Hydroxide‐Based Dentifrice as an Anti‐Erosive Agent in an in Situ Intrinsic Erosion Model.” American Journal of Dentistry 30, no. Suppl 3: 137–141. 29178758

[jemt70042-bib-0035] Passos, V. F. , M. A. Melo , F. F. Silva , L. K. Rodrigues , and S. L. Santiago . 2014. “Effects of Diode Laser Therapy and Stannous Fluoride on Dentin Resistance Under Different Erosive Acid Attacks.” Photomedicine and Laser Surgery 32, no. Suppl 3: 46–151. 10.1089/pho.2013.3629. 24552442

[jemt70042-bib-0036] Passos, V. F. , M. A. S. Melo , J. P. M. Lima , et al. 2018. “Active Compounds and Derivatives of *Camellia sinensis* Responding to Erosive Attacks on Dentin.” Brazilian Oral Research 32: e40. 10.1590/1807-3107bor-2018.vol32.0040. 29846385

[jemt70042-bib-0038] Rabelo, C. S. , J. M. R. Oliveira , I. C. Leal , F. M. L. L. Costa , N. M. P. S. Ricardo , and V. F. Passos . 2023. “The Potential of Galactomannan From Caesalpinia Ferrea on Erosive Dentin Wear Reduction in Vitro.” Brazilian Dental Journal 34, no. Suppl 5: 72–78. 10.1590/0103-6440202305508. PMC10759959 38133475

[jemt70042-bib-0040] Schlueter, N. , A. Hara , R. P. Shellis , and C. Ganss . 2011. “Methods for the Measurement and Characterization of Erosion in Enamel and Dentine.” Caries Research 45, no. Suppl 1: 13–23. 10.1159/000326819. 21625129

[jemt70042-bib-0039] Schlueter, N. , B. T. Amaechi , D. Bartlett , et al. 2020. “Terminology of Erosive Tooth Wear: Consensus Report of a Workshop Organized by the ORCA and the Cariology Research Group of the IADR.” Caries Research 54, no. Suppl 1: 2–6. 10.1159/000503308. 31610535

[jemt70042-bib-0041] Schlueter, N. , J. Klimek , and C. Ganss . 2011. “Efficacy of Tin‐Containing Solutions on Erosive Mineral Loss in Enamel and Dentine in Situ.” Clinical Oral Investigations 15, no. Suppl 3: 361–367. 10.1007/s00784-010-0386-x. 20169458

[jemt70042-bib-0042] Shellis, R. P. , C. Ganss , Y. Ren , D. T. Zero , and A. Lussi . 2011. “Methodology and Models in Erosion Research: Discussion and Conclusions.” Caries Research 45, no. Suppl 1: 69–77. 10.1159/000325971. 21625135

[jemt70042-bib-0043] Skalsky Jarkander, M. , M. Grindefjord , and K. Carlstedt . 2018. “Dental Erosion, Prevalence, and Risk Factors Among a Group of Adolescents in Stockholm County.” European Archives of Paediatric Dentistry 19, no. Suppl 1: 23–31. 10.1007/s40368-017-0317-5. 29327216 PMC5807473

[jemt70042-bib-0044] Toledano, M. , M. Yamauti , M. E. Ruiz‐Requena , and R. Osorio . 2012. “A ZnO‐Doped Adhesive Reduced Collagen Degradation Favouring Dentine Remineralization.” Journal of Dentistry 40, no. Suppl 9: 756–765. 10.1016/j.jdent.2012.05.007. 22659338

[jemt70042-bib-0045] Vargas‐Ferreira, F. , J. R. Praetzel , and T. M. Ardenghi . 2011. “Prevalence of Tooth Erosion and Associated Factors in 11‐14‐Year‐Old Brazilian Schoolchildren.” Journal of Public Health Dentistry 71, no. Suppl 1: 6–12. 10.1111/j.1752-7325.2010.00194.x. 20726945

[jemt70042-bib-0046] Vidal, C. M. , T. R. Aguiar , R. Phansalkar , et al. 2014. “Galloyl Moieties Enhance the Dentin Biomodification Potential of Plant‐Derived Catechins.” Acta Biomaterialia 10, no. Suppl 7: 3288–3294. 10.1016/j.actbio.2014.03.036. 24721612 PMC4041811

[jemt70042-bib-0047] Wang, Y. L. , H. H. Chang , Y. C. Chiang , Y. C. Lu , and C. P. Lin . 2018. “Effects of Fluoride and Epigallocatechin Gallate on Soft‐Drink‐Induced Dental Erosion of Enamel and Root Dentin.” Journal of the Formosan Medical Association 117, no. Suppl 4: 276–282. 10.1016/j.jfma.2018.01.020. 29449065

[jemt70042-bib-0048] West, N. X. , M. Davies , and B. T. Amaechi . 2011. “In Vitro and in Situ Erosion Models for Evaluating Tooth Substance Loss.” Caries Research 45, no. Suppl 1: 43–52. 10.1159/000325945. 21625132

[jemt70042-bib-0049] West, N. X. , T. He , Y. Zou , J. DiGennaro , A. Biesbrock , and M. Davies . 2021. “Bioavailable Gluconate Chelated Stannous Fluoride Toothpaste Meta‐Analyses: Effects on Dentine Hypersensitivity and Enamel Erosion.” Journal of Dentistry 105: 103566. 10.1016/j.jdent.2020.103566. 33383100

[jemt70042-bib-0050] Wiegand, A. , S. Bliggenstorfer , A. C. Magalhaes , B. Sener , and T. Attin . 2008. “Impact of the In Situ Formed Salivary Pellicle on Enamel and Dentine Erosion Induced by Different Acids.” Acta Odontologica Scandinavica 66, no. Suppl 4: 225. 10.1080/00016350802183401. 18607835

[jemt70042-bib-0051] Yan, H. , H. Yang , K. Li , J. Yu , and C. Huang . 2017. “Effects of Chlorhexidine‐Encapsulated Mesoporous Silica Nanoparticles on the Anti‐Biofilm and Mechanical Properties of Glass Ionomer Cement.” Molecules (Basel, Switzerland) 22, no. Suppl 7: 1225. 10.3390/molecules22071225. 28753997 PMC6152133

[jemt70042-bib-0053] Yu, J. , H. Yang , K. Li , H. Ren , J. Lei , and C. Huang . 2017. “Development of Epigallocatechin‐3‐Gallate‐Encapsulated Nanohydroxyapatite/Mesoporous Silica for Therapeutic Management of Dentin Surface.” ACS Applied Materials & Interfaces 9, no. Suppl 31: 25796–25807. 10.1021/acsami.7b06597. 28703572

[jemt70042-bib-0052] Yu, J. , H. Yang , K. Li , J. Lei , L. Zhou , and C. Huang . 2016. “A Novel Application of Nanohydroxyapatite/Mesoporous Silica Biocomposite on Treating Dentin Hypersensitivity: An in Vitro Study.” Journal of Dentistry 50: 21–29. 10.1016/j.jdent.2016.04.005. 27101767

[jemt70042-bib-0054] Zou, R. , S. Gong , J. Shi , et al. 2017. “Magnetic‐NIR Persistent Luminescent Dual‐Modal ZGOCS @ MSNs @ Gd2O3 Core − Shell Nanoprobes for in Vivo Imaging.” Chemistry of Materials 29: 3938–3946. 10.1021/acs.chemmater.7b00087.

